# Leber Hereditary Optic Neuropathy: Review of Treatment and Management

**DOI:** 10.3389/fneur.2021.651639

**Published:** 2021-05-26

**Authors:** Rabih Hage, Catherine Vignal-Clermont

**Affiliations:** Neuro-ophthalmology Department, Hôpital Fondation Rothschild, Paris, France

**Keywords:** Leber's optic neuropathy, idebenone, gene therapy, mitochondrial disease, visual loss

## Abstract

Leber hereditary optic neuropathy (LHON) is a maternally inherited mitochondrial disease that specifically targets the retinal ganglion cells by reducing their ability to produce enough energy to sustain. The mutations of the mitochondrial DNA that cause LHON are silent until an unknown trigger causes bilateral central visual scotoma. After the onset of loss of vision, most patients experience progressive worsening within the following months. Few of them regain some vision after a period of ~1 year. Management of LHON patients has been focused on understanding the triggers of the disease and its pathophysiology to prevent the onset of visual loss in a carrier. Medical treatment is recommended once visual loss has started in at least one eye. Research evaluated drugs that are thought to be able to restore the mitochondrial electron transport chain of the retinal ganglion cells. Significant advances were made in evaluating free radical cell scavengers and gene therapy as potential treatments for LHON. Although encouraging the results of clinical trial have been mixed in stopping the worsening of visual loss. In patients with chronic disease of over 1 year, efficient treatment that restores vision is yet to be discovered. In this review, we summarize the management strategies for patients with LHON before, during, and after the loss of vision, explain the rationale and effectiveness of previous and current treatments, and report findings about emerging treatments.

## Introduction

Inherited mitochondrial diseases (IMDs) are maternally transmitted genetic disorders involving defective cellular energy production. Management and treatment of IMDs remain major challenges for modern medicine due to their rare and heterogeneous nature. Dramatic progress has been achieved in understanding their pathogenesis, but, to date, this has not led to the discovery of a cure.

Leber hereditary optic neuropathy (LHON) is one of the most common IMDs. In 90% of cases, it is caused by one of three primary point mitochondrial mutations. These mutations are clinically silent until some yet unknown trigger induces a respiratory chain dysfunction in the retinal ganglion cells, leading to decreased vision in both eyes (in most cases sequentially, from a few weeks to a few months apart). The loss of vision usually consists in bilateral central scotoma, but the entire visual field can be lost in the most severe cases. Because it involves a specific cell type and permits the administration of treatment during the window of time between the onset of vision loss in the first and second eyes, LHON is a good candidate for clinical trials.

In the last few years, clinical trials for gene therapy as well as idebenone, a synthetic analog of coenzyme Q_10_, have sought to reduce the impact and progression of LHON after its onset. So far, these trials have received most attention. Though encouraging however, the results have been mixed.

In this review, we summarize the management strategies for patients with LHON before, during, and after the loss of vision, explain the rationale and effectiveness of previous and current treatments, and report findings about emerging treatments.

## Pathophysiology and Animal Model

Retinal ganglion cells (RGCs) are neurons located in the inner part of the retina. Their axons bundle around the optic disc and, as they pass through the lamina cribrosa, form the optic nerve. These axons are divided into a myelinated postlaminar part and a non-myelinated prelaminar segment that has higher energy needs and concentration of mitochondria than most, if not all other, neurons. RGCs find their energy in the oxidative phosphorylation (OXPHOS) of nutrients that happens in the mitochondria and produces adenosine triphosphate (ATP). OXPHOS involves several enzymes, including the nicotinamide adenine dinucleotide (NADH):ubiquinone oxidoreductase, also known as NADH dehydrogenase or complex I, which is compounded of several subunits. Three primary point mitochondrial DNA (mtDNA) mutations are found in 90% of LHON patients. These mutations affect different subunits of NADH dehydrogenase, causing dysfunction of the electron transport chain (ETC), decrease in ATP production, and excessive generation of reactive oxidative species (ROS), leading to energy production failure and, eventually, cell death. The result is visual loss in symptomatic patients (1). Beginning with the most frequent, the primary three point mutations are: G11778A, T14484, and G3460A. Since they affect, respectively, subunits 4, 6, and 1 of the NADH dehydrogenase, they are referred to as ND4, ND6, and ND1 mutations. It is virtually impossible to differentiate these mutations clinically at the acute phase of the disease, but the most common one, ND4, has the worst prognosis, with only 4% chance of visual recovery (2). A recent meta-analysis by Newman et al. gathered information about visual outcome in patients with the ND4 mutation from 15 studies (12 retrospective and 3 prospective) (3). They showed that visual recovery, whose definition varied across the studies covered, occurred in 23 out of 204 patients.

Other mutations might be dominant in selected population groups with fewer available genetic data. Non-primary mutations have heterogeneous prognosis, and a limited number of case reports have been published, which prohibit drawing broad conclusions about the severity of each mutation (4).

Visual loss in LHON is directly related to RGC injury and death and, therefore, to the impaired energy production of RGC mitochondria. Research has focused on restoring ATP production in a complex I-deficient mitochondria as well as on identifying the cascade of events that triggers complex I dysfunction. An animal model of complex I deficiency was developed by Zhang et al. (5) in order to test candidate drug treatments. This model consisted in intravitreous injection of a complex I inhibitor (rotenone) in mice's eyes. The effect was consistent with the degeneration of RGC in LHON (5). This animal model has been used to test the neuroprotective strategies discussed in Section Prevention below (6).

## Prevention

Clinicians face two possible scenarios when it comes to a LHON patient. First, the patient has developed recent visual loss and all the medical efforts are focused on reducing its severity. Second, the patient is asymptomatic but knows that he has inherited the mutation from their mother. Here, the clinician's primary concern is to identify the risk factors that can trigger the disease.

However, not all LHON mutation carriers experience loss of vision. Most reports have given penetrances as high as 50–60% in males and 10–20% in females (7–10), although lower penetrances (of 20–27% in males and 4–8% in females) have also been reported in larger cohorts (11). Age is a confounding factor since some late-onset cases have been reported and since younger asymptomatic individuals could have developed symptoms later in their life (12). Why some carriers never develop any symptoms is currently unknown. It is believed that a combination of genetic and environmental influences play a significant role.

### Risk Factors for Visual Loss in LHON

#### Genetic Risk Factors

Penetrance seems not to be related to the type of mutation (11). In the large majority of LHON patients, the mutations are homoplasmic mutants, but the disease has also been reported in heteroplasmic individuals. However, these genetic results should be interpreted with caution since mtDNA in peripheral blood might not reflect the heteroplasmic load in the RGC (13). Finally, it has been shown that the high sequence variability of the mitochondrial genome, which characterizes the different mtDNA haplogroups, modulates the penetrance of IMDs in general, and LHON in particular. Haplotype J is believed to be associated with higher susceptibility to LHON mutations because of a lower amount of mtDNA and mtDNA-encoded polypeptides (14). This might be useful for studying large cohorts but is of little value when it comes to evaluating risk for LHON in individual carriers. At the level of the individual, it has been shown that increased mitochondrial mass protects against the adverse effects of LHON mutation. Giordano et al. (15) have shown in 2014 that mitochondrial mass might explain the variability in penetrance of LHON mutations.

#### Environmental Risk Factors

LHON is characterized by rapidly progressive, though painless, loss of vision with central visual scotoma. This presentation is shared with toxic and nutritional optic neuropathies (TNONs), with the exception that, in the latter, visual loss is experienced simultaneously in both eyes. Because of these common clinical features, toxic substances such as tobacco and alcohol are pointed out as possible triggers for LHON. Discontinuation of intoxication can bring about some visual improvement in TNON. This has led some authors to try tobacco smoke antagonists as a possible treatment for LHON. In 1968, Foulds et al. (16) realized that hydroxocobalamine (vitamin B12) had shown efficiency at treating what was then called “tobacco amblyopia” and decided to treat LHON patients with it. They suspected that the cyanogenic substances contained in tobacco smoke might trigger LHON. Twenty years later, Berninger studied rhodanese activity in LHON patients (17). Berninger thought this mitochondrial enzyme could play a role in the pathophysiology of LHON since it was known to detoxify cyanide by converting it to thiocyanate. Thirteen LHON patients where tested for rhodanese activity, including one at the acute phase of the disease, but none showed abnormal results. Cyanide levels in the blood were also within normal limits.

### Preventive Measures

In 2009, Kirkman et al. conducted a large, multicenter epidemiological study of 196 affected and 206 unaffected carriers of one of the three primary mtDNA mutations. They identified a strong and consistent association between visual loss and smoking, independent of gender and alcohol intake, with a clinical penetrance of 93% in men who smoked (18). The authors concluded that patients should be advised to stop smoking and to reduce their consumption of alcohol.

There is indisputable evidence that tobacco and alcohol play a role in triggering LHON. Other, currently unknown, environmental factors must be implicated since the disease also affects children as well as adults with no history of smoking or drinking. However, childhood-onset LHON has a better prognosis for visual acuity (19).

## Medical Treatment

### Antioxidants

Oxidative stress modulation has been at the center of IMD treatment strategies. The theoretical goal is to increase mitochondrial respiration and reduce ROS that are produced by IMDs. Various molecules have been tried, with different combinations of vitamins (B2, B3, B12, C, E, and folic acid), ubiquinone, and other supplements (e.g., alpha-lipoic acid, carnitine, creatine, l-arginine, glutathion, and dichloroacetate) (20–22). Unfortunately, there is not enough scientific evidence to support their use in LHON.

The ubiquinone family was the most promising treatment. It includes coenzyme Q_10_, an electron shuttle between complex I and complex II of the ETC in the mitochondrial membrane. Coenzyme Q_10_ has been beneficial in other IMDs in which its deficiency causes encephalomyelopathy, but it has not shown improvement in LHON, although a few case reports suggested otherwise (23, 24). A major limitation of coenzyme Q_10_ is its inability to cross the blood–brain barrier when taken orally. To address this issue, idebenone, a synthetic hydrosoluble analog of coenzyme Q_10_, was developed. Idebenone showed a protective effect against complex I-deficient retinal ganglion cell death *in vitro*. It also allowed recovery of vision in rotenone mice (25). The first report of a LHON patient treated with it was published in the Lancet in 1992. A 10-year-old boy with the ND4 mutation was started with 90 mg of idebenone daily, and his vision improved from 6/90 OU to 6/6 in the right eye after 4 months and in the left eye after 7 months (26). Other isolated case reports and small retrospective open-labeled studies have further supported the claim that idebenone might be an effective treatment for improving vision or shortening time to visual recovery in LHON (27–29). In 2011, the “Rescue of Hereditary Optic Disease Outpatient Study” (RHODOS) prospectively randomized 85 patients with LHON with <5 years of visual loss to either a group that received idebenone 900 mg/day or to a group that received a placebo in a double-blinded fashion (30). Treatment lasted 24 weeks; it was found safe and well-tolerated. Primary end point was the best recovery in visual acuity. The study failed to show any significant differences in this regard between the active drug and the placebo. However, a trend was noticed in favor of idebenone when the authors analyzed changes in best visual acuity and excluded patients with the 14488 mutation, known for a higher rate of spontaneous recovery. Two years later, the same team reported persistence of the treatment effect in 60 out of the 85 patients included in the first study (31). The lack of significance of RHODOS might have been linked to the inclusion criteria. Patients with a disease onset as old as 5 years were included. For some of them, optic atrophy had already reached its peak. The likelihood of a positive response was higher among patients with more recent disease onset. In the light of these results, idebenone (Raxone^®^) was approved by the European Medicine Agency to treat LHON in 2015 in adolescents and adult patients at 900 mg/day divided into three doses. As of 2021, a non-interventional study is ongoing, the “Post-Authorisation Safety Study with Raxone^®^ in LHON Patients” (PAROS) study (NCT02771379). It aims to evaluate the long-term safety effectiveness profiles of idebenone when used under conditions of routine clinical care.

### Gene Therapy

#### Bases for Gene Therapy in LHON

In genetic diseases, symptoms are linked to the absence of a protein produced by a gene that is missing or mutated. The goal of gene therapy (GT) is to allow the production of a functional protein by delivering, into a target cell, a copy of a gene that does not include the deleterious mutation. GT uses viral vectors to deliver the desired gene to the nucleus of the target cell. In IMDs, the defective gene is in the mitochondria so the ideal scenario would be to deliver the copy of the gene there. However, this has been impossible, given that mitochondria have a double-membrane structure that constitutes a physical barrier for gene therapy. Furthermore, a higher amount of gene would have to be delivered to a sufficient number of mitochondria in each cell in order to achieve efficacy. Because of these restrictions, a technique called “allotopic expression” has been developed. It consists in functional relocation of mitochondrial genes into the nucleus, followed by import of the gene-encoded polypeptide from the cytoplasm into the mitochondria (32–34).

In LHON, GT consists in delivering a gene to the nucleus of the RGC. This leads to the production, in the cytoplasm or the ribosomes of the RGCs, of a protein that is then redirected into the mitochondria. The goal, more specifically, is to introduce an unmutated MT-ND4 gene into the patient's RGC mitochondria. In 2002, Guy et al. were the first team to use an adeno-associated viral vector (AAVV) to transfect a synthetic ND4 subunit into the mitochondria of cybrids with the ND4 mutation (35). The transfected cells produced three times as much ATP than the mock-transfected cybrids, which the authors interpreted as a successful restoration of complex I-dependent respiration. A series of animal experiments followed, in which rabbit, rat, and murine models received AAVV-mediated gene delivery of human ND4 intravitreously. The different teams showed that injection technique was safe and that there was no ocular complication related to the treatment itself. They also showed mitochondrial internalization of the AAVV, expression of its genetic content, and complementation of the pathogenic phenotype (36–42). Because the ND4 mutation is the most common cause of LHON, GT efforts tend to focus on it. Patients with ND1, ND6, or other LHON mutations are not candidates for gene therapy as of 2021.

#### Clinical Trials

In 2016, Feuer et al. reported the first case series of five legally blind patients with ND4 mutation who were treated with gene therapy in one eye as part of a wider clinical trial meant to evaluate the efficacy of this treatment in LHON. Four out of five participants had visual loss for over 1 year. Three-month follow-up was reported. No patient experienced serious safety events. Two of them showed significant increase in visual acuity (hand motion to seven letters in one and to 15 letters in the second). Interestingly, the patient who had the most dramatic improvement in his visual acuity also showed visual improvement in the contralateral non-injected eye (43). Low and medium doses were used to treat these patients as well as another nine patients whose cases were reported subsequently in 2017 (44). The authors found that the difference in visual acuity between the treated and untreated eye was significantly higher than what they found in their previous natural history study (45). From an anatomical standpoint, there were no significant differences in the thickness of the retinal fiber layers.

GT took a step forward in 2017 with two phase III clinical trials called *rescue* and *reverse* that only differed in the duration of vision loss ( ≤ 6 months for rescue, and >6 months to 1 year for reverse). These studies were randomized, double masked, sham controlled, and multicentric. Included patients had one eye injected with GS010, a recombinant, adeno-associated virus containing a modified cDNA encoding the human wild-type mitochondrial ND4 protein and supporting allotopic expression. The other eye received a sham injection. On average, patients experienced an improvement in their visual acuity of about three lines (15 letters). Surprisingly, a similar improvement of about 13 letters was reported in the other, sham-treated eye. This raised suspicion of transfer of viral vector DNA from the injected eye to the fellow eye, through the optic pathways. A recent non-human primate study demonstrated that this type of transfer is possible (46).

These results are encouraging, but the visual improvement remains limited and variable. GT needs more assessment before it is deemed efficient and is used as a routine care option in LHON. A new clinical trial using GS010 called *reflect* is currently ongoing. In it, patients have both eyes injected with the active drug. Enrollment is over now, and the first results are expected in the next few months.

### Other Medical Treatments

Other treatments than idebenone continue to be used to improve visual acuity in LHON. Reports exist for each modality, and they mostly represent therapeutic interventions that are supported by very few medical teams, if not just one.

#### EPI-743

Alpha-tocotrienol quinone (EPI-743) is a para-benzoquinone that replenishes glutathione stores and has proved to be 1,000 times more efficient than idebenone at reducing oxidative stress *in vitro* (47, 48). This neuroprotective and antioxidant drug demonstrated visual improvement in four out of five LHON patients in whom it was started <4 months after the onset of visual loss (49). EPI-743 has been used in other IMDs, but further investigations are needed to validate its usefulness in LHON (50).

#### Cyclosporine

Cyclosporine A inhibits the opening of the mitochondrial permeability transition pore that plays a crucial role in damage-induced cell death, thereby blocking the apoptosis. Its therapeutic potential in LHON has been evaluated prospectively in five patients with confirmed primary mitochondrial DNA mutations and strictly unilateral optic neuropathy that occurred within 6 months prior to enrollment. Despite treatment with oral cyclosporine A, all patients eventually experienced bilateral eye involvement within 11–65 weeks after the initiation of treatment. Over the study time period, the average best-corrected visual acuity worsened in the first affected eye. By the end of the study, both eyes were equally affected (51).

#### Brimonidine

Brimonidine is an α-2 agonist that is routinely used, as a topical agent, to lower the intraocular pressure in open-angle glaucoma (52). Experimental studies on rats have shown that brimonidine can have a neuroprotective effect on injured optic nerves by reducing apoptosis in cases of elevated intraocular pressure or retinal ischemia (53). In an open-labeled trial, brimonidine was used in nine LHON patients who had recently developed visual loss in one eye. The drug failed to prevent the involvement of the second eye. The authors hypothesized that apoptosis might not be a significant mechanism in RGC death in LHON or that RGC already presented asymptomatic irreversible injury when treatment was started (54).

#### Perspective

New treatment strategies are being considered, but some have never been tested clinically.

- Nutritional interventions are thought to have the potential to enhance mitochondrial bioenergetic capacity. Ketogenic diet (high in fat and low in carbohydrates) mimics the state of ketosis induced by starvation and is thought to have a neuroprotective effect by increasing antioxidant capacity (55, 56).
- The Stem Cell Ophthalmology Treatment Study (SCOTS) included five LHON patients who were given autologous stem cell concentrate through a combination of different modalities (retrobulbar injection, intravitreous injection, vitrectomy with subretinal injection, and intravenously). Three of them had improvement of their vision from hand motion or count finger to a measurable acuity in one eye. However, these three patients did not have the ND4 mutation and were treated at an early stage of their disease. Another study of bone marrow-derived mesenchymal stem cell therapy, SCOTS2, is ongoing. Further evaluation are needed to draw a conclusion on the efficacy of stem cell therapy in LHON (57).
- Mitochondrial biogenesis is a physiological process in which cells increase the mass and number of their mitochondria, allowing greater energy production. Increased mitochondrial biogenesis happens in LHON carriers and is thought to have a protective effect (15). Theoretically, pharmaceutical activation of mitochondrial biogenesis could be used as prevention in carriers, but this complex process is not yet fully understood.
- The gender bias in LHON has led some authors to consider the potential protective value of estrogens and their interactions with RGCs. Pisano et al. have shown that estrogens that bind specifically to receptors located in RGCs can reduce apoptosis in LHON cybrid cells carrying the ND4 mutation (58). The authors concluded that estrogens should be considered a potential preventive measure in carriers and called for a trial in the LHON genetic mouse model.
- Regenerative medicine aims to replace damaged cells. It encompasses several modalities, including induced pluripotent stem cells (iPSCs). In the case of LHON, and most chronic optic neuropathies, the target cells would be the RGCs. Regenerative medicine would be considered when a significant number of RGCs has been lost after the onset of the optic neuropathy. Multiple iPSC lines have been generated from fibroblasts of LHON patients. These lines offer unique opportunities to investigate LHON phenotypes and regulatory mechanisms at the cellular level (59–61). Unfortunately, the generated cells are not organized as they would be in the retina, which prevents their use in cell therapy. 3D approaches that rely on retinal cells other than RGCs are currently under development. Their goal is to allow the iPSC-derived RGCs to be integrated into an environment where they can interact with other constituents of the retinal tissue (62). The greatest challenge of regenerative medicine is ultimately to allow regeneration of the optic nerve itself. To do this, the cells obtained would have to first reach their target location in the retina and then send their axons all the way to the lateral geniculate body, with half of them crossing the optic chiasm along the way (63).
- Mitochondrial replacement therapy (MRT) is a technology that uncouples the inheritance of mtDNA from nuclear DNA. There are several techniques for MRT that differ in the biological form of the nuclear genome when it is transferred within the karyoplast, all of which combine the mother's nuclear DNA with unaffected mtDNA. Hyslop et al. reported the first preclinical study on the pronuclear transfer technique in 2016. This technique consists in transferring the pronuclei from a zygote to another enucleated one. It was shown to restore mitochondrial function even though it only led to a reduction, rather than a complete replacement, of the mutant load (64). In LHON, a technique using iPSC-derived RGCs allowed significant reduction in the level of apoptosis in cybrid-corrected RGCs (65). MRT has never been used to prevent the transmission of a LHON mutation. The first human born from MRT was reported in 2017 by Zhang et al. In that case, MRT was administered to prevent a mitochondrial disease that would have resulted in the *in utero* death of the embryo (66). Because it entails the manipulation of human gametes in a similar way to cloning techniques, MRT raises ethical concerns that still need to be addressed as the technology evolves.

## Supportive Treatment

In LHON, visual impairment can be sudden and dramatic. Few patients recover enough vision to resume a normal life. Thus, LHON is often experienced as an earthquake in the life of an otherwise healthy individual. The lack of efficient treatment leads to situations where patients seek medical advice from multiple specialists, and the main complaints are low vision and inability to cope with simple, everyday tasks. Patients can feel or become dependent as they are unable to mourn the loss of normal vision, as they have experienced it until then. The disease often comes with devastating social cost that caregivers must not overlook.

### Psychological Complications

Reactive depression is common, and a patient's social network can be of great help. However, psychological support must always be offered to all patients after a diagnosis of LHON, whether they are symptomatic or not. In a study about psychological morbidity of LHON, half of the 103 included patients met the depression criteria after vision loss. The authors found that older age was correlated with higher depression prevalence than younger age (67).

### Genetic Counseling

Genetic counseling allows patients to adapt to the psychological and familial implications of LHON. However, the only certainty it gives them is that men will not transmit the mutation to their offspring while women will transmit it to all of theirs. Other variables, such as the risk that siblings of affected individuals will also develop visual loss, are impossible to predict.

### Low Vision Rehabilitation

LHON patients are good candidates for low vision rehabilitation (LVR) when they have a central scotoma and residual peripheral visual field. Patients are taught skills to adapt to their vision and optimize their use of the residual visual field. Reading aids, such as magnifiers or filters, are of great help for improving vision-related quality of life in these patients (68).

## International Consensus Statement

At a conference held in Milan in 2016, a panel of experts from Europe and North America provided consensus statements about therapeutic management of LHON. These statements sought to address the concerns of clinicians and to help in the decision-making process for patient clinical management. There was strong consensus that positive prognostic factors, such as young age or ND6 mutation, do not affect management. The panel concluded that the first-line treatment for non-chronic patients (<1 year since the onset of the disease) should include idebenone at a dose of 900 mg/day for at least 1 year, but that there was no evidence that recommended treatment in the case of chronic ones (over 1 year after the onset in the second eye). For relatives, they recommended lifestyle counseling without treatment (69). At the time, GT was not included in the panel's therapeutic recommendations.

**Figure 1:**
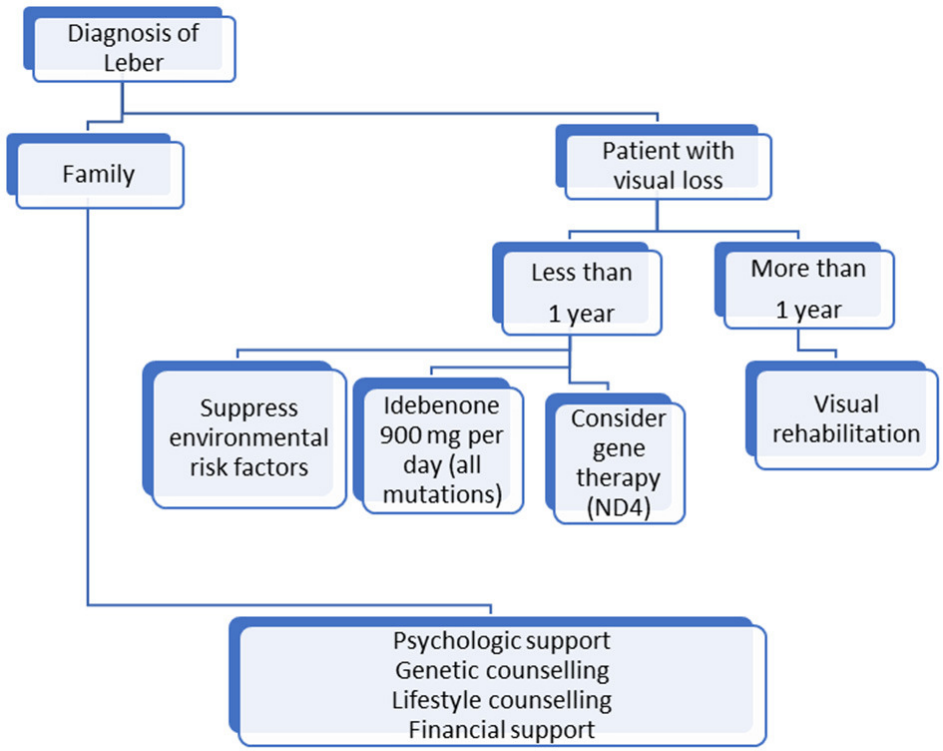
Algorithm for management of patients with Leber optic neuropathy mutation.

Based on these recommendations, we propose a simple algorithm in Figure 1 for LHON patients' management. Research has made great progress but, until an efficient treatment that restores vision is discovered, LHON management must be multidisciplinary and life-long and include prevention and rehabilitation, which remain the only ways to improve patients' quality of life.

## Author Contributions

RH and CV-C contributed equally in the writing of this manuscript. Both authors contributed to the article and approved the submitted version.

## Conflict of Interest

RH and CV-C were investigators in the Gensight Biologics trials RESCUE, REVERSE and REFLECT. RH received financial support from Santhera to attend international meetings in 2018 and 2019. CV-C was a consultant for Santhera and continues to be one for Gensight Biologics.
